# Identification and assessment of cardiolipin interactions with *E. coli* inner membrane proteins

**DOI:** 10.1126/sciadv.abh2217

**Published:** 2021-08-20

**Authors:** Robin A. Corey, Wanling Song, Anna L. Duncan, T. Bertie Ansell, Mark S. P. Sansom, Phillip J. Stansfeld

**Affiliations:** 1 Department of Biochemistry, University of Oxford, South Parks Road, Oxford OX1 3QU, UK.; 2 School of Life Sciences and Department of Chemistry, University of Warwick, Coventry CV4 7AL, UK.

## Abstract

Integral membrane proteins are localized and/or regulated by lipids present in the surrounding bilayer. While bacteria have relatively simple membranes, there is ample evidence that many bacterial proteins bind to specific lipids, especially the anionic lipid cardiolipin. Here, we apply molecular dynamics simulations to assess lipid binding to 42 different *Escherichia coli* inner membrane proteins. Our data reveal an asymmetry between the membrane leaflets, with increased anionic lipid binding to the inner leaflet regions of the proteins, particularly for cardiolipin. From our simulations, we identify >700 independent cardiolipin binding sites, allowing us to identify the molecular basis of a prototypical cardiolipin binding site, which we validate against structures of bacterial proteins bound to cardiolipin. This allows us to construct a set of metrics for defining a high-affinity cardiolipin binding site on bacterial membrane proteins, paving the way for a heuristic approach to defining other protein-lipid interactions.

## Teaser

This study uses MD simulations to identify and analyze >700 binding sites for cardiolipin to a set of bacterial membrane proteins.

## INTRODUCTION

Cells are partitioned and encapsulated by biological membranes that are formed from a complex mixture of different lipids. Here, the lipids provide the necessary hydrophobic environment required to localize and tether the proteins to and/or within the membrane, acting as a solvent for the membrane-spanning region of the protein. In addition, specific interactions between particular membrane lipids and discrete regions on the surface of the protein can be of considerable importance, controlling how the protein folds, localizes, and functions (*1*). Therefore, lipid composition and distribution can have a major impact on the regulation of cell membrane activity.

The identification of specific protein-lipid interactions has been tackled for a number of different proteins. In the well-studied model Gram-negative bacteria *Escherichia coli*, for instance, which has a relatively simple plasma membrane, the anionic phospholipid cardiolipin (“CDL;” also known as “CL”) has been shown to interact specifically with several membrane proteins, including AmtB (*2*), SecYEG (*3*), formate dehydrogenase-N (*4*), and LeuT (*5*–*7*). However, there has been little in the way of systematically modeling CDL interactions with a range of different bacterial proteins in a single study.

Protein-lipid interactions are frequently studied using computational methods, such as with molecular dynamics (MD) simulations (*1*, *8*). These allow analysis of a given protein-lipid interaction with a high spatial and temporal resolution, as well as allowing a relatively unambiguous assignment of molecular species. In particular, the use of a coarse-grained (CG) biomolecular force field, such as Martini (*9*, *10*), has been widely used for studying protein-lipid interactions (*11*). By reducing the degrees of freedom of a given system, sampling is improved, albeit with an associated loss in chemical resolution. This permits the dynamic modelling of protein-lipid interactions, which typically occur on the microsecond time scale.

Here, we use CG simulations to analyze lipid interactions with 42 *E. coli* inner membrane proteins, with each protein simulated in simple bacterial membranes. Global analysis of the data shows a strong bilayer asymmetry, with substantially more anionic lipid binding in the inner leaflet of the membrane, particularly for CDL. This is primarily driven by an increased number of lipid-facing basic residues on the cytoplasmic face of the membrane, extending the well-established positive inside rule (*12*) to residues that interact with the membrane. We then resolve over 700 discrete CDL-binding sites from the dataset and analyze using structural bioinformatics and free-energy calculations. The data allow us to describe rules for a high-affinity CDL-binding site on bacterial membrane proteins. Last, we illustrate that our rules have strong agreement with previously determined CDL sites on bacterial membrane protein structures.

## RESULTS

**Fig. 1:**
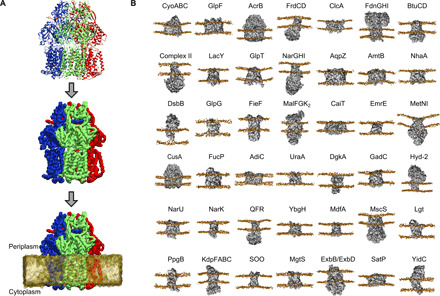
Overview of the methodology. (**A**) Views of an example protein (AcrB; PDB, 1IWG), colored according to chain, shown in the input atomic resolution (top) and in Martini CG description (center) and embedded in a Martini CG lipid membrane (bottom). (**B**) Views of all 42 proteins analyzed in this study, with their common protein names shown above. Protein coordinates are shown in gray, and phosphate beads are shown in orange. PDB and UniProtKB IDs for each system can be found in table S1.

We constructed and simulated 42 different protein/membrane systems of *E. coli* inner membrane proteins using the CG Martini force field [Fig. 1, A and B (*9*, *10*)]. Symmetric membranes were built using 1-palmitoyl-2-oleoyl-*sn*-glycero-3-phosphoethanolamine (POPE), 1-palmitoyl-2-oleoyl-*sn*-glycero-3-phosphoglycerol (POPG), and CDL at a 7:2:1 ratio and simulated for 5 × 5 μs. In total, we generated over 1 ms of simulation data. We used these data to first analyze the global properties of protein-lipid interactions in the model *E. coli* membrane and then to identify and characterize specific protein-CDL interactions.

### Distribution of residues in contact with the membrane

**Fig. 2:**
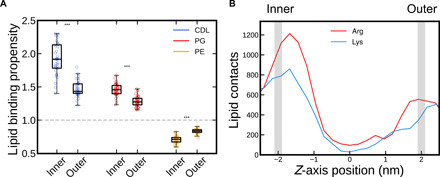
Cross-membrane asymmetry in protein-lipid interactions. (**A**) Quantification of the number of each type of lipid in contact with the different proteins, expressed as a propensity (see Methods). Data are divided between the inner and outer leaflets, with one data point per lipid, per leaflet, per protein. Box plots show the median, upper and lower quartiles, and range (excluding flier points). Statistics are from two-tailed *t* tests, with *P* < 0.001 in all cases. The raw data are plotted in fig. S1A. (**B**) Total number of Arg and Lys residues in contact with lipid molecules, plotted as a function of *z*-axis position, centered on the center of mass of the membrane. Gray lines mark the position of the lipid phosphate groups. Substantially more contacts are made in the inner leaflet than in the outer leaflet. The same analyses for other residues are in fig. S1C.

First, we carried out a global analysis of the nature of protein-lipid interactions in our dataset. Across the 42 systems, we see that CDL, and to a lesser extent PG, binds with a high propensity to the proteins, as measured by quantifying residue-lipid contacts within 0.6 nm (Fig. 2A and fig. S1A). Moreover, there is a strong asymmetry with regard to the inner (cytoplasmic) and outer leaflets, with CDL in particular far more likely to bind the protein when in the inner leaflet of the membrane. Looking at the distribution of residues in contact with the membrane (Fig. 2B), this is explained by both membrane-facing Arg and Lys being substantially more prevalent in the inner leaflet (−2 nm) than in the outer leaflet (+2 nm). This is even more pronounced for CDL-facing residues (fig. S1B). Nonbasic residues are evenly distributed between the two leaflets (fig. S1C). This substantiates that the previously asserted “positive-inside” rule for membrane protein topology (*12*) applies to distribution of not only residues but also amino acids that directly interact with the membrane.

### Analysis of CDL-residue interactions

**Fig. 3:**
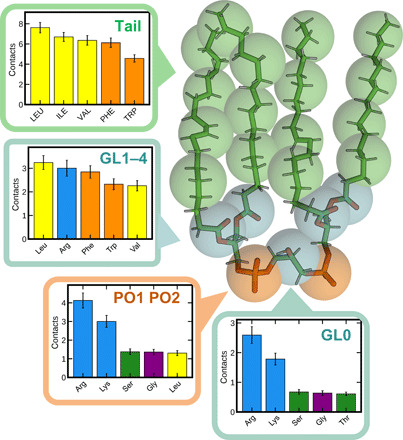
CDL-residue interaction profiles. Number of contacts between each bead type of the Martini CDL molecule and each residue for the proteins analyzed here. The five highest interacting residues are shown. Bar charts show mean and SEM over all 42 systems. Full residue data, and data for PE and PG, are available in fig. S2.

The high binding likelihood of CDL, and the seeming importance of Arg/Lys interactions in this, led us to build interaction profiles for CDL and each residue type. As expected, the CG beads representing the CDL phosphate groups are most likely to be in contact with Arg and Lys residues (Fig. 3), with Arg slightly more prevalent, presumably reflecting the higher propensity for Arg in membrane-facing positions (Fig. 2A). This role of basic residues in CDL binding supports previous structure-based predictions (*13*).

The central glycerol also makes substantial contacts to Arg and Lys (Fig. 3), with Ser, Gly, and Thr residues next most likely. The similarity between the phosphate and glycerol beads is probably due to their close proximity and the shape of the CDL headgroup. The tail-connecting glycerol beads appear to bind to aromatic residues (Phe or Trp), as well as contacting small hydrophobic residues (Leu and Val), or basic residues (Fig. 3).

### Identification of specific CDL-binding sites and the importance of basic residues

**Fig. 4:**
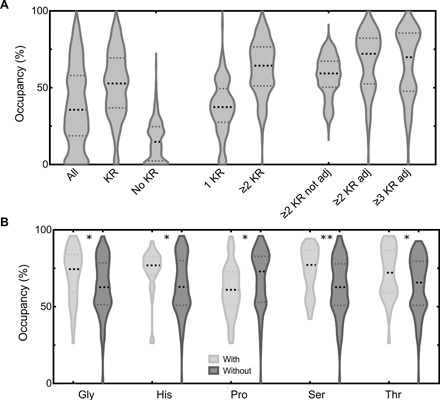
Characterization of identified CDL sites. (**A**) Violin plot showing the computed occupancies for identified CDL-binding sites with binding durations above 10 ns. All sites (“All”), sites with any Arg/Lys residue (“KR”), no Arg/Lys (“No KR”), only one Arg/Lys (“1 KR”), at least two Arg/Lys (“≥2 KR”), and then at least either two or three structurally adjacent Arg/Lys residues (“≥2 KR adj” and “≥3 KR adj”) are shown. Reported median and interquartile range values can be found in table S2. (**B**) CDL occupancies for sites with two or more structurally adjacent Arg/Lys residues and either with or without Gly, His, Pro, Ser, and Thr. Statistical analysis from a two-tailed *t* test, with *P* values of 0.008, 0.007, 0.021, 0.001, and 0.0183, respectively.

We next set out to identify specific CDL-binding sites from our simulation data. We followed an approach described recently (*14*, *15*), where contacts between each residue in the system and each lipid are modeled for every frame of the trajectory, and interaction matrices are constructed. A network analysis clustering protocol is then applied to identify clusters of residues that bind CDL at the same time. For this, only interactions involving the three headgroup beads (GL0, PO1, and PO2) are considered. This analysis was run using a program designed specifically for this purpose (https://github.com/wlsong/PyLipID). From this, we identified 701 specific CDL sites with residence times above 10 ns (see Methods for filtering process). The identified sites had a median of 36% CDL occupancy (Fig. 4A). Representative protein structures with CDL bound are deposited at https://osf.io/gftqa/.

On the basis of the data in Fig. 3, it seems reasonable to predict that the presence of Arg or Lys residues would affect the affinity of the site. Of the 701 sites, ca. 60% contain at least one Arg or Lys residue, and these have a median CDL occupancy of ca. 53% (Fig. 4A), as opposed to just 14% for sites without a basic residue present (Fig. 4A). We also saw a higher number of sites and median occupancy for sites with at least one basic residue in the cytoplasmic leaflet, when compared with the periplasmic leaflet (fig. S3A), although high-affinity periplasmic sites do still exist [see, e.g., (*4*, *16*)].

For the 60% of sites that do contain an Arg or Lys residue, the mean number of basic residues for each site was 1.9 ± 1.3, with an overall site size of six residues (fig. S3B). Hence, we looked at the impact at having two or more basic residues in the site and saw that this gives an even higher site occupancy of 64% (Fig. 4A), as opposed to 37% for only one basic residue (Fig. 4A).

Visualizing some example sites produced by PyLipID reveals that most sites have two to three basic residues in very close proximity to one another (fig. S4). Therefore, we filtered the sites on the basis of the presence of two or more adjacent basic residues (i.e., within 0.8 nm; fig. S3C). Thirty-two percent of sites with basic residues contain adjacent Arg/Lys residues, and typically, these basic residues are very close on the *z* axis (median, 0.21 nm; fig. S3D). Notably, the median occupancy of these sites (72% for two or more basic residues; 70% for three or more) is far higher than that of sites with two or more Arg/Lys residues that are not adjacent (59%; Fig. 4A).

Together, these observations suggest that higher occupancy CDL sites in *E. coli* membrane proteins contain two or more basic residues that are adjacent, i.e., within 0.8 nm, and within 0.2 to 0.3 nm on the *z* axis, i.e., parallel to the membrane.

### Other features of a two–basic residue site

Of the 138 identified sites with adjacent Arg/Lys residues, we analyzed other features that were associated with higher CDL binding. First, analysis of the type of secondary structure the Arg/Lys residues are on reveals that there is no preference for these residues to be either on helix or loop regions of the protein (fig. S5A). This finding contrasts with a previous study looking at mitochondrial structures (*13*), a disparity perhaps explained by the inclusion of loop dynamics in our study.

Then, we looked at other residues present in the binding sites. Several residue types appear to contribute to CDL-binding likelihood, including Gly (ca. 36% of sites), His (ca. 22% of sites), Ser (ca. 30% of sites), and Thr (ca. 27% of sites), which all increase the median occupancy of the CDL site (Fig. 4B). This fits well with the observation that Ser, Gly, and Thr all have high levels of CDL headgroup interactions (Fig. 3). Conversely, Pro (ca. 33% of sites) decreases the median occupancy of the CDL site.

### Contribution of different residues to CDL-binding energy

**Fig. 5:**
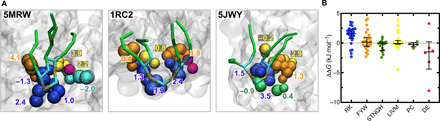
FEP Ala scanning. (**A**) Views of selected CDL sites for which Ala-scanning FEP calculations were run. Each residue is shown in colored spheres, and its calculated contribution to CDL binding is reported as mean (*n* = 10). The bound CDL from the input pose is shown as gold (phosphate), cyan (glycerol), and green (tails) sticks. (**B**) ΔΔ*G* values for each residue type, as calculated using FEP. Each dot is an individual residue to alanine FEP calculation. The median and interquartile range are shown. The values for each residue are broken down in fig. S6C, and the full data are shown in table S3.

To assess the contributions of different residues to CDL binding, we performed alanine-scanning free-energy perturbation (FEP) calculations. Here, a positive ΔΔ*G* value indicates a higher affinity for CDL than PE (see Methods for details). We applied this approach to selected residues in 10 different binding sites, for a total of 102 mutations (see Fig. 5A for three example sites). The data show a reasonable range in the values for each residue type, with the primary observation being that Arg/Lys residues have a median interaction energy of 1.6 (0.8 to 2.4) kJ mol^−1^ for CDL over PE (Fig. 5B), i.e., they interact with CDL more strongly than with PE. Of note, in some cases, the substitution of Arg/Lys for Ala decreases the strength of the CDL interaction. This occurs in cases where there are four or more basic residues in total in the site, suggesting that once two to three basic residues are present, the addition of further basic residues diminishes the strength of the CDL coordination.

In addition, certain aromatic residues show a preference for CDL over PE (Fig. 5B), supporting the prediction in Fig. 3. However, the median interaction energy is close to 0, so these residues need to be assessed with respect to the overall composition of the site.

### CDL-binding site rules and experimental validation

**Fig. 6:**
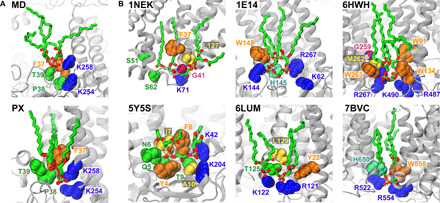
CDL sites from structural studies. (**A**) View of the CDL site from *E. coli* formate dehydrogenase, as determined using MD (top) or structurally [bottom (*4*)]. Note that the top panel is the site as predicted with CG data and converted to atomistic representation for visualization purposes. (**B**) Views of other CDL-binding sites as determined via structural analyses, colored as in Fig. 5. See table S4 for a summary of all 19 identified sites with respect to the proposed CDL-binding rules.

Taking the data together, it is clear that a high-affinity CDL site has a few key features: two to three adjacent basic residues in the same plane of the membrane, one or more polar residues, and a neighboring aromatic residue deeper within the membrane. To evaluate these rules using experimental data, we analyzed structures previously deposited in the Protein Data Bank (PDB). First, a direct comparison of our data with the bound CDL in *E. coli* formate dehydrogenase-N [PDB, 1KQF (*4*)] reveals that our CG data correctly predict the structural site (Fig. 6A), with a very high (74 ± 24%) CDL occupancy across the subunits, and that the site follows the rules outlined above.

We then carried out a broader analysis, identifying a further 18 CDL sites across five additional proteins [from (*17*–*21*); see Methods for details]. We compared these to our CDL site rules, observing excellent agreement (Fig. 6B and table S4).

## DISCUSSION

Membrane proteins bind to, and are often regulated by, many different lipids from the surrounding bilayer. A number of studies have attempted to detect and probe these interactions, usually focusing on one system at a time, with notable exceptions (*11*). Here, we investigate interactions between membrane proteins and lipids in the bacterial inner membrane, focusing on systems for which high-resolution structural data of the *E. coli* membrane protein exist.

Our analyses reveal a notable pattern of asymmetry between the inner and outer leaflets of the membrane, with anionic CDL and PG binding much more readily to the inner leaflet region of the protein. This appears to be driven by an increased number of lipid-facing basic residues on the cytoplasmic face of the proteins (Fig. 2B), as previously predicted (*22*). This might affect the ratios of lipids in each leaflet of the membrane—if CDL and PG are sequestered at high-affinity binding sites on proteins, they are plausibly more likely to avoid recycling, as seen for mitochondrial CDL (*23*), contributing to a net asymmetry between the leaflets of the membrane. It is unclear what the biological necessity for this is: The proposed stabilization of membrane proteins (*24*) and/or role as a proton sink (*25*) in mitochondrial inner membranes could easily act in both leaflets of the membrane. CDL asymmetry does contribute to high membrane curvature in mitochondria (*26*), but this effect is largely absent in *E. coli.* Alternatively, the asymmetry could be in place to help balance the charges arising from the positive inside rule (*12*) or even help to directly establish the positive inside rule by influencing protein topology (*27*). Experimental analyses looking at CDL distribution in the membrane, like those similarly performed for PE distribution (*28*), would be useful to confirm these findings.

In addition, our data reveal a set of rules for a high-affinity CDL-binding site on an *E. coli*—and therefore likely bacterial—membrane protein. These are the following:

1) Two to three basic residues in close proximity, i.e., within 0.8 nm of each other, within 0.2 to 0.3 nm of each other on the *z* axis, and roughly 1.8 nm from the center of the membrane. These likely coordinate the two phosphates of the CDL molecule (Fig. 3). FEP analyses suggest that each basic residue will contribute, on average, 1.6 kJ mol^−1^ to CDL binding above that of PE—and sometimes up to 4 to 5 kJ mol^−1^—and suggest that more than three basic residues is not necessary or desirable for a CDL site.

2) The presence of at least one polar residue, e.g., Ser, Thr, or His. These are often in a similar plane to the basic residues and are likely important for stabilizing the CDL headgroup, particularly the central glycerol.

3) One or more aromatic residues, slightly deeper within the membrane. These probably coordinate the glycerol groups connecting the phosphate headgroup to the acyl tails.

We also note the common occurrence of Gly residues at the CDL headgroup, which is associated with a higher binding affinity of CDL (Fig. 4B). The lack of a side chain might help Gly pack tightly against the central glycerol of CDL, although additional analysis with atomistic simulation would be necessary to confirm this.

CDL is also highly abundant and functionally important in the mitochondrial membrane, where it has been shown to bind specifically to a wide range of proteins, including Tim23/Tim50 (*29*), F-ATPase (*30*), and Complex I (*31*). Previous structure-based analyses of mitochondrial proteins suggest overlap with the rules we identify here (*13*, *32*). It would therefore be interesting to extend these analyses to mitochondrial proteins to see how universal our proposed CDL-binding rules are.

Certain caution should be drawn from the use here of a CG model of CDL. This reduces the accuracy with which interactions are defined, particularly in terms of electrostatics, which will affect basic residues, and polarizability, which will particularly affect aromatic side chains. However, considerable success has been achieved using CG to model protein-lipid interactions (*1*, *8*), and here, we use the newest version of Martini (v3), which should have an improvement in accuracy (*33*). Moreover, direct comparison of the CG and atomistic binding poses for a chosen system suggests that good agreement is retained at the atomistic resolution (fig. S7).

Future work incorporating additional atomistic data will permit additional insight into the data presented here and allow a higher degree of accuracy when distinguishing between similar sites with different lipid binding properties. Nevertheless, the increased chemical resolution will also likely make data interpretation more difficult, necessitating the use of more advanced statistical analyses.

While here we have largely focused on CDL over PG binding, there may be interesting comparisons to be made between these two anionic lipids in terms of how they interact with the different binding sites. While the headgroups are similar, i.e., two PG molecules is roughly one CDL, there are differences that arise from the central glycerol of CDL holding the two charged phosphate groups in close proximity. To bring two PG headgroups this close within a binding site would have both an enthalpic (Coulombic) and an entropic cost, which is not the case for CDL. Furthermore, the CDL has a very specific headgroup structure that it seems unlikely that two PG molecules would readily adopt.

Our study focuses principally on lipid headgroups, with little analysis of the contribution of lipid tails to binding. As a necessary simplification, we chose to use simple palmitoyl-oleyl tails, where oleyl was chosen to represent the bacterial vaccenyl tail group. Therefore, future analyses might also be important to investigate lipid tail diversity (*34*).

## METHODS

### Building systems

We referred to the MemProtMD database [http://memprotmd.bioch.ox.ac.uk (*35*, *36*)] to identify 42 unique *E. coli* inner membrane proteins with structural information available in the PDB. For each protein, a single representative set of coordinates was chosen, with the full list of PDB IDs used found in table S1. For each PDB ID, the atomic coordinates of the protein were downloaded from the MemProtMD database. The protein coordinates were extracted and converted to the Martini 3 open beta package v3.0.b.3.2 (*9*, *10*, *33*). All side chains were set to their default charge state, with His set to neutral. The proteins were then built into symmetric *E. coli* inner membranes using the insane protocol (*37*) with 67% POPE, 23% POPG, and 10% CDL (using a −2 charge model with 23 CG beads; see Supplementary Methods for the topology used) in each leaflet. Note that because of the CG nature of the Martini force field, here, oleyl has been chosen to represent to common bacterial vaccenyl tail group.

Systems were solubilized with Martini 3 waters and ions to a neutral charge. Systems were minimized using the steepest descent method and then equilibrated in two rounds using 5-fs time steps for 1 ns and 20-fs time steps for 100 ns. Both equilibration steps used a semi-isotropic Berendsen barostat (*38*) at 1 bar and a velocity-rescaling thermostat (*39*) at 323 K. Production simulations were then run using the Parrinello-Rahman barostat (*40*) at 1 bar using 20-fs time steps over 5 μs, running five repeats. All simulations were run using Gromacs 2019 (*41*, *42*).

The systems were analyzed using gmx tools and MDAnalysis (*43*). Images were made with VMD (*44*), and plots were made with Matplotlib (*45*) and Prism 8.

### Modeling asymmetry in lipid contacts

For each of the 42 protein systems, the total number of each lipid type in contact with the protein was determined for both inner and outer leaflets of the membrane, as determined using the topology information present in the Orientations of Proteins in Membranes (OPM) database (*46*). For each of the five repeats, average contacts (based on the distance between any protein residue and any bead from the lipid molecule being less than 0.6 nm) were taken for 0.5 to 5 μs of each simulation, using the Gromacs tool gmx select. Data for CDL, PG, and PE binding to each protein were combined and plotted as lipid binding propensity, where propensity is defined as

$$\frac{\text{Target lipid as}\;\%\;\text{of bound lipid}}{\text{Target lipid as}\;\%\;\text{of total lipid}}$$

So, if 20% of the lipid bound to the protein surface was CDL, and 10% of the total lipid was CDL, then the propensity would be the ratio of these, i.e., 20/10 = 2. The raw data are plotted in fig. S1A.

### Position of lipid-contacting residues across the membrane

As the vast majority of the systems had planar bilayers, to establish a profile for protein-lipid interactions across the span of the membrane, simulations were aligned according to the lipid phosphate beads such that the center of the membrane was set to 0 nm on the *z* axis. The probability that each residue in the system contacts any lipid over the 5 × 5 μs of data was then calculated on the basis of a 0.6 nm cutoff. For every residue with a lipid contact probability greater than 10% of the simulation time, we extracted the *z*-axis position from the final frame of the PO4 bead normalized simulation. We then plotted a histogram of these residues along the *z* axis.

### CDL-residue interactions

Predictions of CDL-binding sites were made on the basis of the frequency of contact of each CDL particle with different protein residues across all 42 systems. Contact was determined as the number of frames of the simulation, where the specified particles from the lipid and residue were within 0.6 nm, calculated using MDAnalysis. For each bead type, the five highest contacting residue types were plotted, with all residues plotted in fig. S2 for all three lipid types.

### Identification of lipid binding sites

Identification of CDL-binding sites was performed following a kinetic analysis of residue-lipid interactions, based on (*14*, *15*). The program we wrote for this purpose is available at https://github.com/wlsong/PyLipID, with full details to be published separately. In brief, our approach determines whether each possible lipid/residue pair is in contact at each frame of the simulation and then uses graph theory to cluster residues with high likelihood of simultaneously binding the same CDL headgroup. For this, a double cutoff model is used: Once the lipid-residue distance is smaller than the first cutoff of 0.55 nm, it is considered bound until the distance goes over a distance of 1 nm. A dual cutoff is used to account for variability in the lipid position within the binding site due to random fluctuations. Only CDL was analyzed, and only interactions involving the three headgroup beads (GL0, PO1, and PO2) were analyzed.

For each site, a global occupancy of the site was calculated on the basis of the number of frames that CDL spends in contact with at least one residue in the site (frames_bound_)

$$\text{Occupancy}=\frac{{\text{frames}}_{\text{bound}}}{{\text{frames}}_{\text{total}}}$$

From the 42 systems, we identified 986 CDL-binding sites, with binding site residence times (the time the lipid is continuously in contact with any residue from the site) ranging from extremely short (ca. 1-ns time scale) to 2 to 3 μs in length. To simplify our data, we chose only the 701 sites with calculated binding site residence times above 10 ns, as any time below this threshold is likely just accounted for by random diffusion of the CDL molecule. The inclusion of these sites does not affect the main outcomes of the study (e.g., fig. S3E). In addition, any individual residues with binding occupancies below 10% of the total site occupancy were removed before analysis.

### Identification of adjacent basic residues in sites

To determine which sites contain adjacent basic residues, we extracted the Cartesian coordinates of the backbone (“BB”) bead of all Arg or Lys residues from the identified site from the input model. If an individual site has two or more basic residues within 0.8 nm in three-dimensional space, we classified these as an adjacent pair. The value 0.8 nm was chosen as a reasonable cutoff based off the distribution of the distance between basic residues in sites with two or more basic residues present (fig. S3C).

### Alanine scanning FEP

For selected identified sites with CDL occupancies above 50%, as determined using PyLipID, alanine scanning FEP calculations were performed on any residue in contact with the CDL for at least 50% of the overall site occupancy, apart from Gly and Ala (being too similar to Ala). For this, the selected residues were alchemically perturbed to the one-bead Martini 3 open beta Ala through conversion of their side-chain (SC) beads to dummy particles with no Lennard Jones (LJ) or Coulombic interactions. We ran these FEP calculations in the presence or absence of CDL to measure the effect of mutation on lipid binding [as per (*47*)].

Poses for each site comprising the protein and bound CDL were produced using the PyLipID program. These were then embedded into a solvated Martini POPE membrane, using the insane protocol. The systems were minimized using steepest descents and equilibrated for 10 ns using 20-fs time steps, as described above. The lipid was kept in the binding site using a 1000 kJ mol^−1^ nm^−2^ flat bottom restraint between the center-of-mass (COM) of the CDL headgroup and the COM of the site residues, applied using plumed 2.2.3 (*48*, *49*). For calculations of the system without CDL, the CDL molecule was deleted, and a 100-ns equilibration simulation was run to allow the membrane to equilibrate around the protein.

For the FEP calculations, Coulombic and LJ parameters were switched separately over the λ coordinate, over 17 windows with certain windows overlapping, following this scheme:

; init_lambda 0 1 2 3 4 5 6 7 8 9 10 11 12 13 14 15 16

vdw_lambdas = 0.0 0.0 0.0 0.0 0.0 0.0 0.0 0.1 0.2 0.3 0.4 0.5 0.6 0.7 0.8 0.9 1.0

coul_lambdas = 0.0 0.1 0.2 0.3 0.4 0.5 0.6 0.7 0.8 0.9 1.0 1.0 1.0 1.0 1.0 1.0 1.0

Each λ window was run for 10 repeats for 12 ns, with the first 2 ns discarded as equilibration. The separate windows were constructed into energy landscapes along λ using Multistate Bennett Acceptance Ratio (MBAR) (*50*) as implemented in alchemical analysis (*51*). Convergence is shown for one case (5JWY) in fig. S6A.

ΔΔ*G* values were computed using the cycle in fig. S6B. Here, we determined the energy cost of substituting a residue to an alanine when bound (Δ*G*_arg>ala.CDL_) and not bound (i.e., in a pure PE membrane; Δ*G*_arg>ala.PE_) to a CDL molecule. A positive ΔΔ*G* (Δ*G*_arg>ala.CDL_ − Δ*G*_arg>ala.PE_) means that the residue is interacting more strongly with the CDL than with a generic lipid.

### Analysis of PDB entries

The PDB was queried for the chemical ID “CDL,” giving 222 structures (as of February 2021), 64 of which were bacterial. In addition, one system in which the ligand was labeled as “CDN” was added. Filtering out duplicate entries for the same system left seven unique structures, with 19 CDL sites. Comparison with the proposed CDL rules was made on the basis of visual inspection (see table S4). Note that PDBs containing modified fluorescent CDL derivatives were not included in this analysis.

### Atomistic simulations

For a CG snapshot of CDL bound to formate dehydrogenase N from our FEP analyses, we converted the system to an atomistic description using the CG2AT (v2) protocol (*52*). Protein and lipids (1 CDL and 848 POPE) were described with the CHARMM36 force field (*53*) and solvated with TIP3P water and Na^+^ and Cl^−^ to 150 mM.

The systems were energy-minimized using the steepest descents method and then equilibrated with positional restraints on heavy atoms for 100 ps in the NPT ensemble at 310 K with the V-rescale thermostat and semi-isotropic Parrinello-Rahman pressure coupling. A production simulation was run without positional restraints, with 2-fs time steps over 200 ns.

## SUPPLEMENTARY MATERIALS

Supplementary material for this article is available at http://advances.sciencemag.org/cgi/content/full/7/34/eabh2217/DC1

## Appendix

View/request a protocol for this paper from *Bio-protocol*.
